# Functional Connectivity Disruption in Subjective Cognitive Decline and Mild Cognitive Impairment: A Common Pattern of Alterations

**DOI:** 10.3389/fnagi.2017.00109

**Published:** 2017-04-21

**Authors:** David López-Sanz, Ricardo Bruña, Pilar Garcés, María Carmen Martín-Buro, Stefan Walter, María Luisa Delgado, Mercedes Montenegro, Ramón López Higes, Alberto Marcos, Fernando Maestú

**Affiliations:** ^1^Laboratory of Cognitive and Computational Neuroscience, Center for Biomedical Technology, Complutense University of Madrid and Technical University of MadridPozuelo de Alarcón, Spain; ^2^Department of Basic Psychology II, Complutense University of MadridPozuelo de Alarcón, Spain; ^3^Centro de investigación biomédica, Getafe HospitalGetafe, Spain; ^4^Memory Decline Prevention Center Madrid Salud, Ayuntamiento de MadridMadrid, Spain; ^5^Neurology Department, San Carlos Clinical HospitalMadrid, Spain

**Keywords:** Alzheimer disease, mild cognitive impairment, magnetoencephalography, Subjective Cognitive Decline, Functional connectivity

## Abstract

Functional connectivity (FC) alterations represent a key feature in Alzheimer's Disease (AD) and provide a useful tool to characterize and predict the course of the disease. Those alterations have been also described in Mild Cognitive Impairment (MCI), a prodromal stage of AD. There is a growing interest in detecting AD pathology in the brain in the very early stages of the disorder. Subjective Cognitive Decline (SCD) could represent a preclinical asymptomatic stage of AD but very little is known about this population. In the present work we assessed whether FC disruptions are already present in this stage, and if they share any spatial distribution properties with MCI alterations (a condition known to be highly related to AD). To this end, we measured electromagnetic spontaneous activity with MEG in 39 healthy control elders, 41 elders with SCD and 51 MCI patients. The results showed FC alterations in both SCD and MCI compared to the healthy control group. Interestingly, both groups exhibited a very similar spatial pattern of altered links: a hyper-synchronized anterior network and a posterior network characterized by a decrease in FC. This decrease was more pronounced in the MCI group. These results highlight that elders with SCD present FC alterations. More importantly, those disruptions affected AD typically related areas and showed great overlap with the alterations exhibited by MCI patients. These results support the consideration of SCD as a preclinical stage of AD and may indicate that FC alterations appear very early in the course of the disease.

## Introduction

The increase in life expectancy during the last decades has an inherent downside: the increase of dementia cases in the population (Brookmeyer et al., 2007). AD is the most common cause of dementia, accounting for ~60–80% of cases (Barker et al., 2002; Wilson et al., 2012), therefore its early diagnose has become a relevant public health issue. Early interventions at an early stage of the disease before the appearance of extensive brain lesions are the most promising approach for reducing the burden of dementia in the population (Imtiaz et al., 2014).

The description of Mild Cognitive Impairment (MCI), an intermediate state between healthy individuals and dementia patients, represented an important step toward the early diagnose of AD. MCI is characterized by slight cognitive impairment in one or more domains and increased risk of developing dementia (Petersen, 2004). The presence of AD-pathology in MCI patients is a very consistent finding (Tabatabaei-Jafari et al., 2015; Villemagne and Chételat, 2016), and the annual conversion rate from MCI to AD has been estimated around 8–15% (Petersen, 2016). Over the last few years there is a growing interest in what has been suggested to be a pre-clinical asymptomatic stage of AD: Subjective Cognitive Decline (SCD; Jessen et al., 2014). Accordingly, SCD could represent a prodromal stage of MCI in which individuals report a worsening in their cognitive skills that current neuropsychological assessment tools are not able to capture. SCD subjects are at a greater risk for developing MCI or AD compared to healthy elders (Jessen et al., 2010, 2014; Reisberg et al., 2010; Mitchell et al., 2014). Whilst there are still inconsistent results, some neuroimaging studies reported AD-related brain pathology in SCD supporting its interpretation as an asymptomatic stage of AD as reviewed by Sun et al. (2015).

Among the variety of neuroimaging tools available, the International Working Group has recently highlighted the great sensitivity of functional connectivity (FC) EEG/MEG measures in the very early stages of AD, and the limited number of electrophysiological studies on this stage (Dubois et al., 2016). FC disruption in AD is a consistent finding in the literature (Gili et al., 2011; Teipel et al., 2016). In fact AD has been described as a disconnection syndrome (Delbeuck et al., 2003). Moreover, these abnormalities have also been reported for MCI patients (Sorg et al., 2007; Bai et al., 2009) and have demonstrated great utility in predicting future cognitive decline and conversion to AD (Petrella et al., 2011; López et al., 2014). However, the knowledge about the course of FC alterations in the very early stages of AD is really scarce, and the few available results in fMRI resting state are inconsistent (Hafkemeijer et al., 2013; Wang et al., 2013). The only study to date using MEG in SCD reported a decrease in FC during the performance of a memory task (Bajo et al., 2012), but resting state FC was not addressed.

In this study, we reconstructed for the first time source space whole brain resting state functional networks with MEG of healthy elders, SCD and MCI patients in the alpha band. A previous study from out group demonstrated that SCD elders exhibit spectral alterations specific to this frequency range (López-Sanz et al., 2016). Furthermore, results in this frequency range have demonstrated great consistency in previous works studying MCI and AD patients (Jelic et al., 2000; Adler et al., 2003; Babiloni et al., 2006). Most of the studies reported a prominent decrease in FC in both MCI and AD patients. The study of other frequency bands reached somewhat inconsistent results (for a review, see Babiloni et al., 2015). Hence, we hypothesized that the onset of the FC cascading failure in AD takes place in the SCD stage, thus SCD patients will show an intermediate FC pattern of alterations between those exhibited by controls and MCI sharing some spatial properties. Additionally, in order to help the interpretation and comparison of our results with previous literature we conducted FC analysis in two key resting state networks (RSN) consistently associated with AD: default mode network (DMN) and dorsal attention network (DAN; Greicius et al., 2004; Li et al., 2012).

## Materials and methods

### Subjects

The study sample was recruited from three different centers: the Neurology Department in “Hospital Universitario San Carlos,” the “Center for Prevention of Cognitive Impairment,” and the “Seniors Center of Chamartin District” located in Madrid (Spain).

The sample consisted of 131 right-handed and native Spanish speakers. Fifty-one of them were diagnosed as mild cognitive impaired (MCI group), while 80 showed no objective neuropsychological impairment. The latter were further divided in two groups, 39 healthy control elders without any cognitive concern (HC group) and 41 with subjective cognitive decline (SCD group). Table 1 summarizes their demographic data and other relevant characteristics.

**Table 1:** **Demographic, neuropsychological and neurophysiological data for each group**.

	**Mean** ± ***SD***			***p*****-values**		
	**HC**	**SCD**	**MCI**	**HC–SCD**	**HC–MCI**	**SCD–MCI**
Age	70.4 ± 3.7	71.6 ± 4.5	73.0 ± 3.7	n.s.	5.3·10^−3^	n.s.
Gender (M/F)	1.7 ± 0.5	1.8 ± 0.4	1.6 ± 0.5	n.s.	n.s.	n.s.
GDS	0.9 ± 1.1	1.4 ± 1.2	2.7 ± 2.1	n.s.	5.9·10^−7^	2.3·10^−4^
MMSE	29.0 ± 1.1	28.9 ± 1.1	27.4 ± 2.0	n.s.	1.7·10^−6^	5.8·10^−6^
Direct digits	8.5 ± 1.9	8.8 ± 2.1	7.1 ± 2.1	n.s.	2.8·10^−3^	2.5·10^−4^
Inverse digits	6.2 ± 2.1	5.7 ± 2.0	4.4 ± 1.5	n.s.	1.1·10^−5^	3.0·10^−3^
BNT	53.0 ± 8.7	50.9 ± 6.3	44.7 ± 8.6	n.s.	4.7·10^−6^	7.9·10^−4^
Hippocampal volume	5.0·10^−3^ ± 0.5·10^−3^	5.0·10^−3^ ± 0.7·10^−3^	4.4·10^−3^ ± 0.7·10^−3^	n.s.	5.8·10^−6^	4.0·10^−5^

*The left half of the table shows mean ± SD (standard deviation) of demographic information, some neuropsychological scores and neurophysiological data for each group. The right half of the table shows the p-values resulting of the ANOVA comparisons between groups. If the comparison is not significant (p > 0.05) the value is replaced by n.s. (non-significant). GDS stands for Geriatric Depression Scale—Short Form. MMSE stands for Mini Mental State Examination. BNT stands for Boston Naming Test*.

### Diagnostic criteria

To assess the general cognitive and functional status the following set of screening questionnaires were used: The Mini Mental State Examination (MMSE; (Lobo et al., 1979), the Geriatric Depression Scale–Short Form (GDS; Yesavage et al., 1982); the Hachinski Ischemic Score, (HIS; Rosen et al., 1980), and the Functional Assessment Questionnaire (FAQ; Pfeffer et al., 1982).

After the initial screening all subjects underwent an exhaustive neuropsychological assessment including: Direct and Inverse Digit Span Test (Wechsler Memory Scale, WMS-III), Immediate and Delayed Recall (WMS-III), Phonemic and Semantic Fluency (Controlled oral Word Association Test, COWAT), Ideomotor Praxis of Barcelona Test, Boston Naming Test (BNT), Trail Making Test A and B (TMTA and TMTB; Reitan, 1958) and Rule Shift Cards (Behavioral Assessment of the Dysexecutive Syndrome, BADS).

MCI group subjects were diagnosed as MCI according to the criteria established by Petersen (2004) and Grundman (2004). MCI patients did not fulfill criteria for dementia diagnosis.

Cognitive concerns were self-reported by the participants in an interview with clinician experts. The final group assignment was made after neuropsychological evaluation attending to a multidisciplinary consensus (by neuropsychologists, psychiatrists, and neurologists). In order to prevent possible confounders of SCD, problematic medication, psycho-affective disorders or other relevant medical condition lead to the exclusion from the study. Following the recommendations made by the SCD-I-WG, all subjects were older than 60 at onset of SCD, and the onset of SCD occurred within the last 5 years.

The exclusion criteria employed in this study were the followings: (1) history of psychiatric or neurological disorders or drug consumption that could affect MEG activity, such as cholinesterase inhibitors; (2) evidence of infection, infarction or focal lesions in a T2-weighted scan within 2 months before MEG acquisition; (3) a modified Hachinski score equal or higher to 5; (4) a GDS-SF score equal to or higher to 5; (5) history of alcoholism, chronic use of anxiolytics, neuroleptics, narcotics, anticonvulsants or sedative hypnotics. All participants were between 65 and 80 years old at the moment of the MEG acquisition. Besides, additional analyses were conducted to rule out other possible causes of cognitive decline such as B12 vitamin deficit, diabetes mellitus, thyroid problems, syphilis, or Human Immunodeficiency Virus (HIV).

All participants signed an informed consent prior to study enrollment. This study was approved by the “Hospital Universitario San Carlos” ethics committee, and the procedures were performed in accordance with approved guidelines and regulations.

### MEG recordings

Neurophysiological data was acquired by using a 306 channel (102 magnetometers, 204 planar gradiometers) Vectorview MEG system (Elekta AB, Stockholm, Sweden), placed inside a magnetically shielded room (VacuumSchmelze GmbH, Hanau, Germany) at the “Laboratory of Cognitive and Computational Neuroscience” (Madrid, Spain). All recordings were obtained while subjects were sitting comfortably, resting awake with eyes closed. MEG acquisition consisted of 4 min of signal for each subject.

Head shape was obtained by using a three-dimensional Fastrak digitizer (Polhemus, Colchester, Vermont). Three fiducial points (nasion and left and right preauricular points) and at least 300 points of the surface of the scalp were acquired for each subject. In addition, four head position indication (HPI) coils were placed on the subjects scalp, two in the mastoids and two in the forehead. HPI coils' position was also acquired using the Fastrak device, and continuous head position estimation was used during the recording in order to track head movements. Finally, a vertical electrooculogram of the left eye was used to capture blinks and eye movements.

MEG data was acquired using a sampling rate of 1,000 Hz and an online anti-alias bandpass filter between 0.1 and 330 Hz. Recordings were processed offline using a spatiotemporal signal space separation algorithm (Taulu and Simola, 2006); correlation window 0.9, time window 10 s in order to remove magnetic noise originated outside the head. The algorithm was also used to correct head movements of the subject during the recording.

### MEG signal preprocessing

Ocular, muscular and jump artifacts were first identified using an automatic procedure from the Fieldtrip package (Oostenveld et al., 2011), and then visually confirmed by a MEG expert. The remaining data was segmented in 4 s epochs of artifact-free activity. Subjects with at least 15 clean epochs were selected for further analysis (47.6 ± 7.3 epochs in the HC group, 46.2 ± 9.4 epochs in the SCD group, 42.2 ± 7.0 epochs in the MCI group, mean ± standard deviation). In addition, an ICA-based procedure was employed to remove the electrocardiographic component when it was clearly identified. Due to data redundancy after the spatiotemporal filtering, only magnetometers data were used in the subsequent analysis.

### MRI acquisition

A T1-weighted MRI was available for each subject, acquired in a General Electric 1.5 Tesla magnetic resonance scanner, using a high-resolution antenna and a homogenization PURE filter (Fast Spoiled Gradient Echo sequence, TR/TE/TI = 11.2/4.2/450 ms; flip angle 12°; 1 mm slice thickness, 256 × 256 matrix and FOV 25 cm). MRI images were processed with Freesurfer software (version 5.1.0) and its specialized tool for automated cortical and subcortical segmentation (Fischl et al., 2002) in order to obtain the volume of several brain areas. Hippocampal volumes were selected as anatomical evidences of brain atrophy characteristic for MCI and AD (Dubois et al., 2007), and normalized with respect to the overall intracranial volume (ICV) to account for differences in head volume over subjects.

### Source reconstruction

The source model consisted of 2,459 sources placed in a homogeneous grid of 1 cm in MNI template, then linearly transformed to subject space. Each source was labeled as belonging to one of the 64 areas of the reduced Harvard-Oxford atlas (Desikan et al., 2006). Of the initial 2,459 sources, 970 were discarded due to not being identified as part of any recognized area (i.e., white matter sources). The leadfield was calculated for the remaining 1,489 sources using a three-shell Boundary Element Method (brain-skull, skull-scalp and scalp-air interfaces generated from the subject's T1 MRI) model computed with OpenMEEG software (Gramfort et al., 2010).

Source reconstruction was performed for the alpha band. In order to calculate alpha band in the most representable and comprehensive way for our sample we visually identified the individual alpha frequency (IAF) for every participant as the most prominent alpha peak in the average power spectrum over occipital and parietal channels. The average IAF in the study sample was 9.4 Hz. Then, according to Klimesch considerations about alpha band width (Klimesch, 1999), we set it from 6.9 to 11.4 Hz (i.e., IAF-2.5 Hz − IAF + 2 Hz).

Artifact-free epochs were bandpass filtered in the alpha band using an 1,800 order FIR filter designed using Hanning window. Data was filtered in a two-pass procedure to avoid phase distortion, and using 2,000 samples of data at each side as padding to avoid edge effects. Lastly, a Linearly Constrained Minimum Variance (LCMV) beamformer (Van Veen et al., 1997) was employed to obtain the source time series by using the computed leadfield and the epoch-averaged covariance matrix.

### Connectivity analysis

The analysis of FC was performed using the hypothesis of phase synchronization (Rosenblum et al., 2001) and evaluated using the Phase Locking Value (PLV; Lachaux et al., 1999). PLV is calculated using the instantaneous phase difference between a pair of signals. For each temporal point, a vector is constructed with norm unity and phase equal to the phase difference between the instantaneous phases of both signals. Then, PLV-value for each data segment is calculated as the norm of the average vector for the pair of signals *k* and *l*:

$$PLV_{k,\;l}=\left|\frac{1}{T}\sum_{t}e^{-j\left(\varphi_{k}\left(t\right)-\varphi_{l}\left(t\right)\right)}\right|$$

where φ_*k*_(*t*) is the instantaneous phase of signal *k* at instant *t*, and *T* the number of temporal points per segment.

In order to reduce the dimensionality of the connectivity matrices, PLV-values are averaged over areas, obtaining a unique PLV-value for each pair of areas defined in the reduced Harvard-Oxford atlas. The final PLV-value between areas *A* and *B* is calculated as follows:

$$PLV_{A,\;B}=\frac{1}{N_{A}N_{B}}\sum_{N_{A}}\sum_{N_{B}}\left|\frac{1}{T}\sum_{t}e^{-j\left(\varphi_{A_{k}}\left(t\right)-\varphi_{B_{l}}\left(t\right)\right)}\right|$$

where *N*_*A*_ is the number of sources in area *A*, and *A*_*k*_ is the source *k* inside this area.

**Figure 1:**
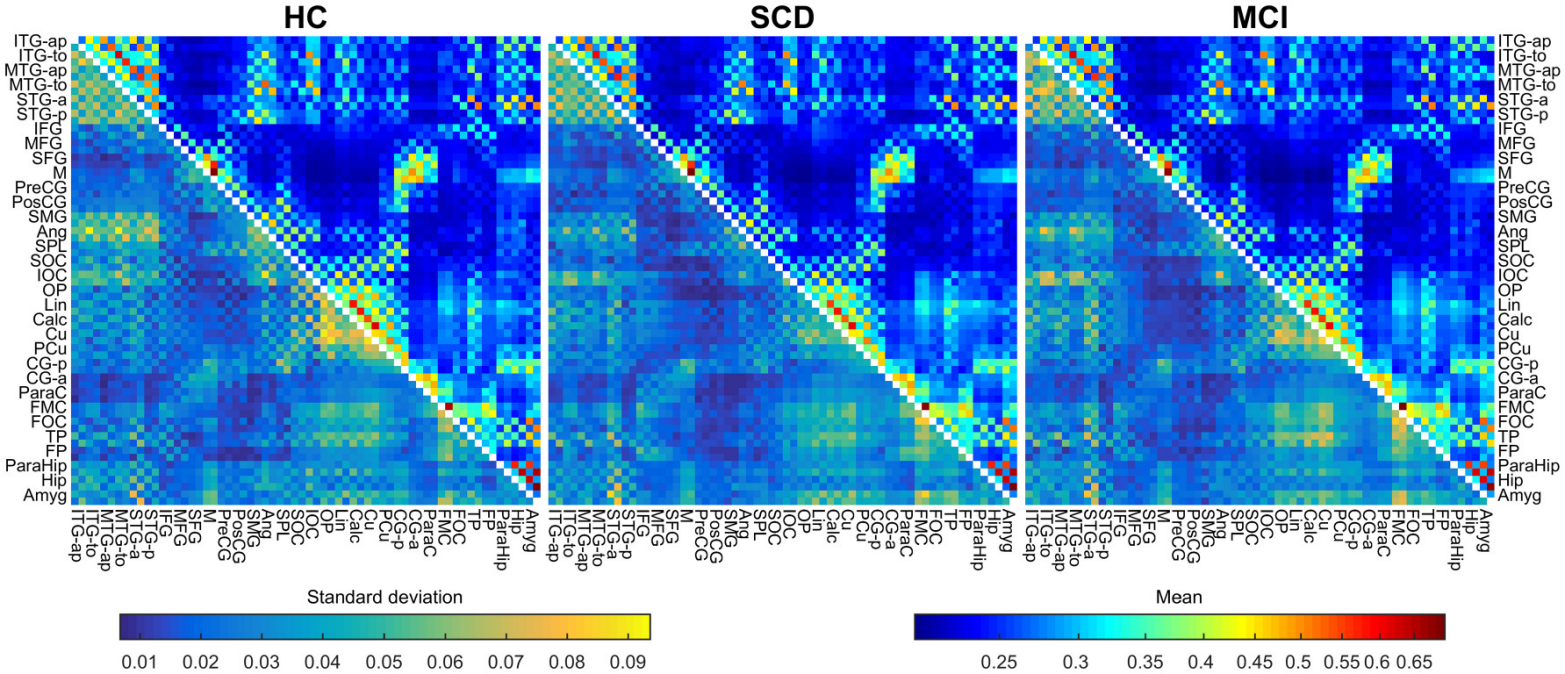
**Mean and standard deviations of the connectivity values for HC (left)**, SCD **(middle)**, and MCI **(right)** groups. The upper triangular area of each matrix shows the mean connectivity value for each link, using a logarithmic scale. The lower triangular area of each matrix shows the standard deviation for each link, using a linear scale. Metrics use different color maps to avoid confusion.

In this work the instantaneous phases of the signals were obtained using the Hilbert analytical signal, with 2,000 samples of data at each side as padding in order to avoid edge effects. Mean and standard deviation FC matrices for each group are shown in Figure 1.

We checked the significance of each individual link for each group separately to ensure results interpretability using the analytical method depicted in (Wilkie, 1983; García-Prieto et al., 2017). All the links were significant after FDR correction (*Q* = 0.05) in the three diagnostic groups (all *p* < 0.028 for HC, all *p* < 0.033 for SCD and all *p* < 0.033 for MCI). Furthermore, in order to evaluate whether between-groups FC differences could be explained by source leakage, the Pearson correlations between beamformer weights were computed for each link and compared across groups (Garcés et al., 2014).

### Network calculation

We calculated the FC in two key RSN: DAN and DMN. The coordinates of the nodes forming each RSN were defined in MNI space according to previous work (Watanabe et al., 2013; Jimenez et al., 2016). In turn, ROIs belonging to the DMN were subdivided into an anterior component (aDMN) and a posterior component (pDMN; Andrews-Hanna et al., 2007). The representative FC for each RSN was computed by averaging the FC-values over all pairs of regions comprising the corresponding network.

### Statistical analysis

In order to assess the differences in connectivity between groups we used an ANCOVA analysis with age as covariate. To ensure the accuracy of the results the ANCOVA analysis was non-parameterized using permutations. The original *F*-value obtained with the ANCOVA analysis and the original grouping were stored, and a series of 10,000 randomizations of subjects were performed, preserving the original group sizes. For each randomization a new *F*-value was obtained, which allowed creating a null-distribution of *F*-values adapted to the characteristics of the data. This distribution was used to calculate the permutation-corrected *p*-value associated to the original *F*-value.

Due to the high number of comparisons, of the order of 2,000, the probability of obtaining an elevated number of false positives is high. In order to avoid this problem we complemented the ANCOVA analysis with a False Discovery Rate (FDR; Benjamini and Hochberg, 1995) multiple comparisons correction, using a *Q* = 0.05 (a 5% rate of false positives).

Finally, for those links who survived the FDR correction we performed a pairwise comparison analysis with Tukey's Honestly Significant Difference correction to determine the groups in which the connectivity values were significantly different. These results were permutation-corrected in a similar way that the ANCOVA results.

Average FC-values for each RSN were compared across groups employing a similar procedure with ANCOVA analysis non-parametrized using permutations.

We performed an ANOVA statistical test with FDR, using a *Q*-value of 0.05, to identify and discard those links whose connectivity value is below the random threshold. Regarding the beamformer weights correlation, we employed a similar procedure to identify links whose correlation between beamformer filters differed among groups.

Last, we conducted correlation analysis in our sample using Pearson coefficient to characterize the relationship between the observed FC changes and cognitive state, as well as hippocampal volume. In order to address the multiple comparisons problem, all *p*-values were also corrected using FDR.

## Results

### Connectivity values

**Figure 2:**
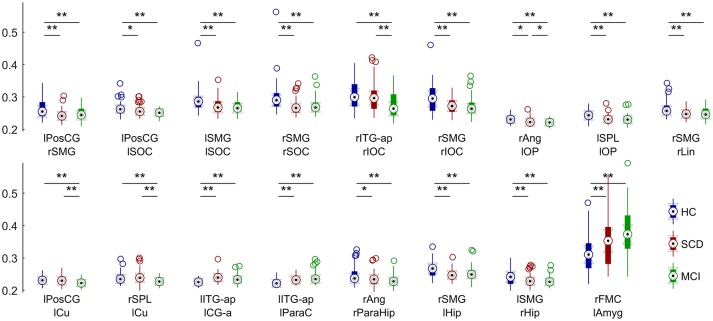
**Distribution of connectivity values in each significantly different link for HC (blue), SCD (red) and MCI (green) groups**. Asterisks mark significantly different connectivity values between groups. One asterisk (^*^) denotes a *p*-value between 0.05 and 0.01. Two asterisks (^**^) denote a *p*-value lower than 0.01.

The FC analysis in this work was performed between anatomical areas, using a reduced version of the Harvard-Oxford atlas, consisting on the regions and abbreviations shown in Table 2. All the links were included in the analysis, as none of them was discarded because of a non-significant PLV-value. The ANCOVA analysis brought significant between-group differences for 17 links in the overall comparison, FDR (*Q* = 0.05) corrected (Figure 2). When observed in the pairwise comparison, the results showed a posterior network, with decreased connectivity, and an anterior network, with increased connectivity for both SCD and MCI compared to the HC group.

**Table 2:** **List of ROIs of the anatomical atlas**.

**Abbreviation**	**Full name**
Amyg	Amygdala
Ang	Angular Gyrus
Calc	Calcarine cortex
CG	Cingulate Gyrus
Cu	Cuneal Cortex
FMC	Frontal Medial Cortex
FOC	Frontal Orbital Cortex
FP	Frontal Pole
Hip	Hippocampus
IOC	Inferior Lateral Occipital Cortex
ITG	Inferior Temporal Gyrus
ITG	Inferior Frontal Gyrus
Lin	Lingual Gyrus
M	Motor cortex
MFG	Middle Frontal Gyrus
MTG	Middle Temporal Gyrus
OP	Occipital Pole
ParaC	Paracingulate Gyrus
ParaHip	Parahippocampal Gyrus
PCu	Precuneous
PosCG	Postcentral Gyrus
PreCG	Precentral Gyrus
SFG	Superior Frontal Gyrus
SMG	Supramarginal Gyrus
SOC	Superior Lateral Occipital Cortex
SPL	Superior Parietal Lobule
STG	Superior Temporal Gyrus
TP	Temporal Pole

**Figure 3:**
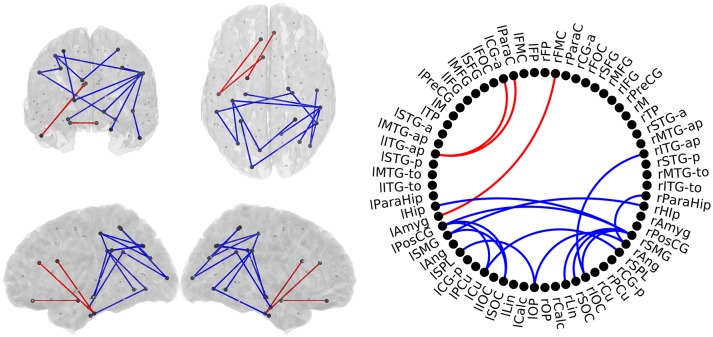
**Links with significantly different FC-values in the comparison between Healthy Controls (HC) and Mild Cognitive Impairment (MCI) groups. Left** : Posterior, superior, left, and right views of the brain. **Right**: Circle plot shows a schematic view of the significant links. Red lines indicate an increased FC-value in MCI respect to HC. Blue lines indicate a decreased FC-value in MCI respect to HC.

*Correspondence between abbreviations showed in Figures 1–3 and regions depicted in the Harvard-Oxford anatomical atlas (Desikan et al., 2006). Preceding letter l or r stands for left or right hemisphere, respectively. Abbreviations ending in -a, -p, -ap, or -to stand for anterior part, posterior part, antero-posterior part, or temporo-occipital part, respectively*.

### HC vs. MCI

In this comparison between HC subjects and MCI patients, all 17 links showed significant group effect in the pairwise comparison (*p* < 0.05; Figure 3). The anterior network presented higher FC in the MCI group compared to the HC group in three links connecting anterior regions, including left Inferior Temporal Gyrus, left Paracingulate and left Anterior Cingulate. The posterior network exhibited lower FC in the MCI group, and comprised 14 links between connecting posterior cortical structures such as: temporal medial structures (both hippocampi and right parahippocampus), parietal (left Postcentral Gyrus, both Supramarginal Gyri), and occipital areas (left Frontal Pole, both Superior Occipital cortices, right Inferior Occipital Cortex, right Lingual Cortex).

### HC vs. SCD

**Figure 4:**
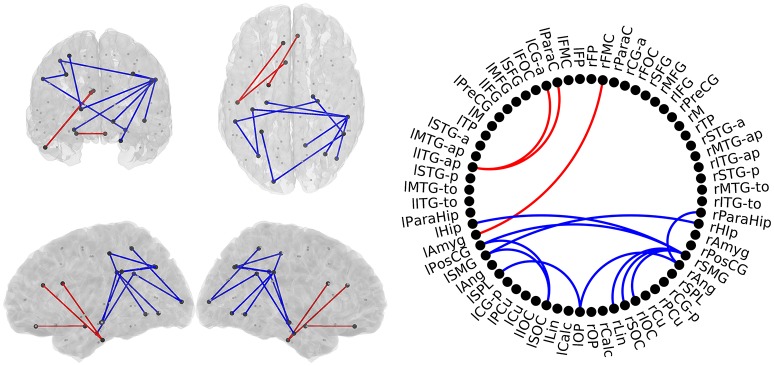
**Links with significantly different FC-values in the comparison between Healthy Controls (HC) and Subjective Cognitive Decline (SCD) groups**. Left: Posterior, superior, left and right views of the brain. Right: Circle plot shows a schematic view of the significant links. Red lines indicate an increased FC-value in SCD respect to HC. Blue lines indicate a decreased FC-value in SCD respect to HC.

In this comparison we identified a similar pattern with two sub-networks, as shown in Figure 4. SCD subjects showed increased FC-values respect to the HC group (*p* < 0.05) in three links connecting the same regions described in the previous comparison. SCD subjects also showed decreased FC in 11 links (*p* < 0.05). Those links connected both intra- and inter-hemispherical areas between posterior regions. Interestingly, all the links affected in the SCD group, were also disrupted in the MCI group in a similar manner.

### SCD vs. MCI

**Figure 5:**
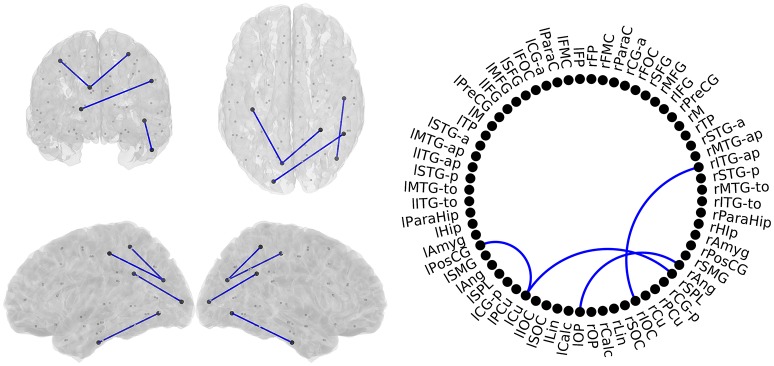
**Links with significantly different FC-values in the comparison between Subjective Cognitive Decline (SCD) and Mild Cognitive Impairment (MCI) groups**. Left: Posterior, superior, left and right views of the brain. Right Circle plot shows a schematic view of the significant links. Blue lines indicate a decreased FC-value in MCI respect to SCD.

MCI patients showed a network comprising four links where FC-values were significantly lower (*p* < 0.05) compared to SCD FC-values (Figure 5). This network with reduced FC connected temporal, parietal and occipital regions of the brain, and comprised both intra- and inter-hemispheric links.

### Differences in RSN

**Figure 6:**
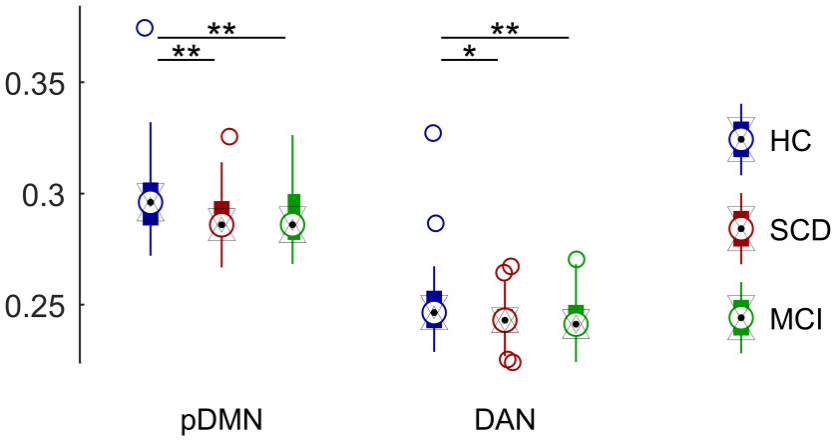
**Distribution of mean connectivity values for each significantly different resting state network for HC (blue), SCD (red), and MCI (green)**. Asterisks mark significantly different connectivity values between groups. One asterisk (^*^) marks a *p*-value between 0.05 and 0.01. Two asterisks (^**^) mark a *p*-value lower than 0.01.

Regarding DAN we observed a significant group effect (*p* < 0.05). Pairwise comparisons revealed a FC decrease in DAN with respect to HC group in both SCD (*p* < 0.05) and MCI (*p* < 0.01). Mean FC of the DAN was not significantly different between SCD and MCI (Figure 6).

DMN was divided into two sub-networks and compared separately. We did not observe any significant group effect for the aDMN component. On the contrary, we found a significant group effect in pDMN (*p* < 0.005). FC was significantly decreased in both SCD (*p* < 0.005) and MCI (*p* < 0.005) with respect to control. SCD and MCI did not show significant differences in mean FC in this sub-network (Figure 6).

### Correlation of beamformer weights

The correlation analysis between the beamformer weights determined there were no differences in the beamformer filters weights of the three groups. Taking this result into account, it is unlikely that the difference in the connectivity metrics could be caused by source leakage (Garcés et al., 2014).

### Differences in hippocampal volume

Due to its extended use in the clinical diagnosis of MCI and AD, we completed this work by analyzing the hippocampal volumes of the participants. The corrected hippocampal volumes were compared between groups by means of an ANCOVA using the age as covariate. The contrast revealed significant differences between groups (*p* < 0.01; Table 1). Pairwise comparisons showed that MCI patients had significantly lower volumes than both SCD (*p* < 0.01) and HC group (*p* < 0.01), while the volumes of SCD and HC participants did not differ (*p* > 0.05).

### Correlation analyses

Some of the links with a reduction in FC in SCD and MCI exhibited positive associations with hippocampal volume, MMSE, inverse digits test and BNT. This means that those subjects with lower FC-values demonstrated an overall worse cognitive status, performed worse in working memory, executive functioning and language, and had smaller hippocampi. On the other hand, all three links with increased FC in SCD and MCI showed negative associations with BNT performance, meaning that those subject with larger FC hyper-synchronization in these specific links performed worse in language and executive functioning. All significant correlations and links are shown in Table 3.

**Table 3:** **Results of the correlation analysis between connectivity values and neuropsychological scores**.

	**Hippocampal volume**	**MMSE**	**Inverse digits**	**BNT - Spontaneous responses**
lPosCG	*n.s*.	*n.s*.	*n.s*.	*n.s*.
rSMG				
lPosCG	*r* = 0.2817	*n.s*.	*n.s*.	*n.s*.
lSOC	*p* = 0.0012			
lSMG	*r* = 0.2504	*r* = 0.2262	n.s.	*r* = 0.2350
lSOC	*p* = 0.0041	*p* = 0.0094		*p* = 0.0076
rSMG	*n.s*.	*r* = 0.2567	*n.s*.	*n.s*.
rSOC		*p* = 0.0031		
rITG-ap	*n.s*.	*n.s*.	*n.s*.	*r* = 0.2783
rIOC				*p* = 0.0015
rSMG	*n.s*.	*n.s*.	*n.s*.	*n.s*.
rIOC				
rAng	*n.s*.	*n.s*.	*r* = 0.2411	*n.s*.
lOP			*p* = 0.0059	
lSPL	*n.s*.	*n.s*.	*r* = 0.3003	*n.s*.
lOP			*p* = 0.0005	
rSMG	*n.s*.	*n.s*.	*n.s*.	*n.s*.
rLin				
lPosCG	*n.s*.	*n.s*.	*n.s*.	*n.s*.
lCu				
rSPL	*n.s*.	*n.s*.	*n.s*.	*n.s*.
lCu				
rAng	*n.s*.	*n.s*.	*n.s*.	*n.s*.
rParaHip				
rSMG	*n.s*.	*n.s*.	*n.s*.	*r* = 0.2651
lHip				*p* = 0.0025
lSMG	*n.s*.	*n.s*.	*n.s*.	*r* = 0.2343
rHIp				*p* = 0.0078
lITG-ap	*n.s*.	*n.s*.	*n.s*.	*r* = −0.3118
lCG-a				*p* = 0.0003
lITG-ap	*n.s*.	*n.s*.	*n.s*.	*r* = −0.3615
lParaC				*p* = 0.0000
rFMC	*n.s*.	*n.s*.	*n.s*	*r* = −0.2602
lAmyg				*p* = 0.0030

*Correlation values of each significant correlation between the significantly different links and the anatomical values and neuropsychological scores. First 14 rows depict hypo-synchronization links. Last three rows depict hyper-synchronization links. The signification values were FDR corrected (Q = 0.05) and the significance threshold was subsequently placed at p = 0.0094. n.s. indicates non-significant correlations. MMSE stands for Mini Mental State Examination. BNT stands for Boston Naming Test*.

## Discussion

In the present work, we have showed that elders presenting with SCD, in spite of their normal neuropsychological performance, exhibit a pattern of FC alterations similar to that shown by MCI patients. In addition, FC alterations were not restricted to DMN regions; they also affected broader areas largely unexplored in previous literature. When compared to the HC group, MCI patients exhibited a marked decrease in FC in the alpha band over posterior areas accompanied by an increased FC in anterior-ventral regions of the brain. The spatial distribution of alterations exhibited by the SCD group mimicked MCI patients' network disruption. According to our results, disconnection among posterior brain regions is even more pronounced in MCI patients when compared to SCD elders. These results draw a continuum in the preclinical phases of the disease where connectivity disruptions would take origin in the very early phases of AD (SCD stage), characterized by initial anterior FC increases and posterior FC decreases, followed in later stages by further posterior decreases in FC. Additionally, we obtained significant correlations of these FC changes with cognition and hippocampal volume, highlighting their pathological nature. To the best of our knowledge, this is the first time that whole brain FC impairment in the alpha band is reported in SCD patients. These results suggest that a subjective feeling of cognitive worsening with a preserved cognitive function could represent an early indicator of preclinical AD pathology in the brain.

It should be noted that most of the existing literature addressing FC in AD has focused specifically on DMN, and more work is needed to characterize whether those findings are specific to DMN areas (Zarahn et al., 2007). We found decreased FC in SCD and MCI involving several areas classically related to the DMN, such as angular gyrus, medial temporal structures (i.e., hippocampus and parahippocampal cortex) or lateral inferior parietal areas. These connectivity decreases over DMN critical areas have been extensively reported (Sorg et al., 2007; Zarahn et al., 2007; Hsiao et al., 2013). In addition, MCI patients showed decreased FC between parietal regions and occipital regions consistent with recent findings reporting decreased FC between DMN regions and visual network (Cai et al., 2016). Beyond marked decreases among posterior cortical regions, MCI patients also showed increased FC over anterior-ventral areas, involving temporal and frontal structures. Initial reports demonstrated that aging was associated to posterior decrements in FC and unspecific anterior increases and decreases (Jones et al., 2011). In this same study, AD patients exhibited a greater FC decrease over posterior regions and only increases among anterior brain regions, which is in line with our findings in the MCI and SCD groups. This anteroposterior dual pattern of hyper- and hypo-connectivity, respectively, has been described as a common feature of the network failure starting in pre-dementia stages and progressing along the AD continuum (Jones et al., 2015).

The most novel and relevant finding of our study is the disruption in brain FC in SCD participants. This pattern of alterations seems to be consistent with findings in AD literature. Our analysis revealed a posterior disconnection over lateral inferior parietal, medial temporal and occipital areas in SCD elders compared to the HC group. Furthermore, SCD participants exhibited an anterior hyper-synchronization affecting the exact same regions where MCI patients showed hyper-synchronization. To date, there are very limited yet contradictory results about the evolution of FC in SCD (Hafkemeijer et al., 2013; Wang et al., 2013), and no study has addressed resting state FC with MEG or EEG. However, our results seem to support previous literature in at-risk of AD populations describing similar anterior-ventral elevated FC and posterior diminished FC in prodromal stages of AD (Brier et al., 2012) and in cognitively normal APOE ε4 carriers (Machulda et al., 2011).

Interestingly, when comparing SCD and MCI groups, we found decreased FC in MCI subjects between occipital, inferior temporal and parietal regions. These results highlight the progressive FC loss throughout AD different stages, consistent with the conception of AD as a disconnection syndrome occurring along a continuum (Delbeuck et al., 2003). Furthermore, the localization of this progressive disconnection is consistent with previous work proving, with a very large sample, that posterior DMN subsystem connectivity declines linearly throughout the course of the disease (Jones et al., 2015). Additionally, our results add value extending these findings to other brain regions not limited to DMN, and more importantly, to the very early preclinical stages of the disease in the SCD and MCI stage. It is worth noting that the anterior hyper-synchronization observed in SCD and MCI groups did neither progress nor decrease from SCD to the theoretically subsequent MCI stage. This is again in line with the above-mentioned work and others (Damoiseaux et al., 2012). Anterior hyper-synchronization across the entire AD spectrum appeared to have a trend toward declining in later stages of the disease. Our results seem to suggest that this increase in FC observed in early AD could actually take place in the preclinical stage of the disease, before clinical symptoms onset, to then slope toward MCI and early AD stages before the subsequent decline in FC already described in late AD.

The clinical impact of the AD-related anterior hyper-synchronization has been highlighted over the past few years. Traditionally, these alterations have been interpreted as a compensatory mechanism (Mormino et al., 2011). Alternatively, it has been proposed that cortical hubs, while acting as critical nodes of the network may augment the pathological cascade in AD increasing the deposition of Aβ (Buckner et al., 2009). In the same vein, these hyper-connected regions could represent the fingerprint of noisy or inefficient synaptic transmission. This synaptic burden could be propagated to downstream networks leading to what has been termed as a cascading network failure in AD (Jones et al., 2015). In fact, higher levels of Aβ accumulation can be caused by increases in synaptic activity (Cirrito et al., 2008). Furthermore, previous MEG results showed that hyper-synchronization of anterior cingulate with certain brain regions predicted a fast conversion from MCI to AD (López et al., 2014). In our work, SCD and MCI anterior alterations involved areas classically related to the salience network. Interestingly, FC increases in these brain regions have been linked with mood disorders in mild AD patients (Balthazar et al., 2014). The association of SCD and certain psychiatric conditions has represented a controversial topic in the field (Buckley et al., 2013). However, cognitive concerns along with these affective symptoms could represent a common susceptibility toward AD as has been already suggested (Snitz et al., 2015). The pathological nature of this hyper-synchronization is reinforced by our correlation results revealing that those subjects with higher synchronization values in these links performed worse in an executive function and language task.

After exploring whole brain FC we focused on two RSN's crucial to AD progression and understanding. Attentional networks (ventral and dorsal) are known to be impaired in AD (Li et al., 2012). However, we studied DAN as it was the only one consistently disturbed in MCI patients in previous literature (Qian et al., 2015; Zhang et al., 2015). Our analysis revealed a significant decrease in the mean FC of DAN in MCI patients supporting previous results. However, our results suggest that this decrease seems to occur in an early stage, when elders still present only cognitive concerns, and maintain this level of hypo-synchronization at least until MCI stage. Alterations in DAN has been related to deficits in top-down attentional control in AD (Zhang et al., 2015).

DMN is considered the key RSN in AD progression. In fact, Aβ spread has been topologically linked to this network (Grothe et al., 2016). The role of posterior cingulate cortex (PCC) in AD progression has received major attention, as it is a crucial hub of pDMN, and it is often used as a seed for DMN studies. The vast majority of previous studies agree that PCC is progressively disconnected from other brain regions inside DMN, reporting FC decreases in AD and MCI patients with either fMRI (Toussaint et al., 2014; Weiler et al., 2014; Kim et al., 2016), a combination of DTI and a functional technique (Soldner et al., 2012; Garcés et al., 2014) or with EEG (Hsiao et al., 2013). However, literature is not without some controversy, as some studies reported an increase in directional connectivity from PCC to other posterior brain regions in MCI. Our results support the majority of studies reporting a decrease in FC over pDMN in the AD-continuum. According to our results, FC alterations start in SCD phase, and remain constant along MCI stage. Of note, we did not observe any significant change in aDMN, which is in line with some previous work (Song et al., 2013; Kim et al., 2016), but not all (Jones et al., 2015). This discrepancy could be explained by the pathological stage of the sample studied, which usually consists of AD patients, thus in a more severe phase of the disease than our sample. Additionally, it has been mentioned that the antero-posterior FC dual change pattern could not be limited to DMN regions (Wiepert et al., 2017), which looks to be confirmed by our results.

FC deficits have been previously linked to structural abnormalities in MCI patients (Garcés et al., 2014). Although we did not address structural connectivity, we observed a significant decrease in hippocampal volume in our MCI group. Noteworthy, hippocampal volume correlated positively with FC in two hypo-synchronized links, in a way that those subjects with smaller hippocampi showed more aberrant FC-values. No changes in hippocampal volume were observed in SCD elders, underscoring the ability of MEG FC to detect subtle brain changes before structural alterations become evident.

This work constitutes the first report of whole-brain FC alterations in SCD with a high temporal resolution technique such as MEG. These FC disruptions affected AD typically-related areas and showed great overlap with MCI alteration pattern, but with a milder intensity. Furthermore, DAN and pDMN were affected in both SCD and MCI, showing a similar level of FC decrease. This is in agreement with previous neuroimaging findings reporting a continuum in the preclinical stages of AD, namely a relatively progressive increase in the burden of certain biomarkers increasing from healthy aging, to SCD and then MCI such as reduced metabolism (Scheef et al., 2012) or brain atrophy (Saykin et al., 2006). Future work should address the predictive value of these alterations, and test whether FC disruption represents a hallmark of AD pathology, increasing the likelihood of MCI and SCD elders to develop further stages of the AD.

## Ethics statement

All participants signed an informed consent prior to study enrollment. This study was approved by the “Hospital Universitario San Carlos” ethics committee, and the procedures were performed in accordance with approved guidelines and regulations.

## Author contributions

DL, RB, and PG, analyzed the data. DL and RB wrote the manuscript and prepared figures. All authors participated in the design of the research, the acquisition of the data and critically reviewed this work.

### Conflict of interest statement

The authors declare that the research was conducted in the absence of any commercial or financial relationships that could be construed as a potential conflict of interest.
